# Imaging Neural Plasticity following Brain Injury

**DOI:** 10.1155/2017/2083294

**Published:** 2017-02-28

**Authors:** Lijun Bai, Lin Ai, Kevin K. W. Wang

**Affiliations:** ^1^The Key Laboratory of Biomedical Information Engineering, Ministry of Education, Department of Biomedical Engineering, School of Life Science and Technology, Xi'an Jiaotong University, Xi'an 710049, China; ^2^Athinoula A. Martinos Center for Biomedical Imaging, Department of Radiology, Massachusetts General Hospital, Harvard Medical School, Charlestown, MA, USA; ^3^Department of Nuclear Medicine, Beijing Tiantan Hospital, Capital Medical University, Beijing, China; ^4^Program for Neurotrauma, Neuroproteomics and Biomarker Research, Departments of Psychiatry and Neuroscience, University of Florida, Gainesville, FL, USA

The human brain possesses a superior capacity to reorganize and profound plasticity after focal lesions following brain injury such as trauma, ischemia, and degenerative disorders. The concept of plasticity describes the mechanisms that rearrange cerebral organization following a brain injury. The development of sophisticated noninvasive neuroimaging techniques over the past decade provides a unique opportunity to examine brain plasticity in humans and invaluable insights into the mechanisms underlying neuroplasticity. Unifying pathogenesis of brain injury by neuroimaging techniques can be beneficial to develop therapeutic strategies with broad applicability for disease prevention and an opportunity to decrease morbidity and mortality from these disorders in human beings.

In this special issue on imaging neural plasticity following brain injury, we compiled a series of articles that represent novel primary research and explore brain plasticity following brain injury. T. Wang et al. (“Impairments in brain Perfusion, Metabolites, Functional Connectivity, and Cognition in Severe Asymptomatic Carotid Stenosis Patients: An Integrated MRI Study”) investigate the brain impairments following asymptomatic carotid stenosis by utilizing an integrated MRI including pulsed Arterial Spin Labeling (pASL) 22 MRI, Proton MR Spectroscopy (MRS) and resting-state functional MRI (R-fMRI). They found that hypoperfusion in the left frontal lobe, lower NAA/Cr ratio in the left hippocampus, and decreased connectivity to the posterior cingulate cortex in the anterior part of the default mode network might partly contribute to the cognition impairment in these patients.

X. Fan et al. (“Distinctive Structural and Effective Connectivity Changes of Semantic Cognition Network across Left and Right Mesial Temporal Lobe Epilepsy Patients”) explore the distinctive brain structural and effective connectivity changes within the semantic cognition network by comparing left and right mesial temporal lobe epilepsy (mTLE) patients and these patients to matched healthy controls. Since seizure attacks were rather targeted than random for patients with hippocampal sclerosis (HS), gray matter atrophy of left mTLE was more serve than that of right mTLE across the whole brain and especially within the contralateral semantic cognition network. This study suggested that left HS patients had a higher vulnerability to seizure attacks, reflecting the compensation strategy. The altered effective connectivity between subregions of the ATL may be the possible reason to explain the more severe name-finding impairment but good comprehension ability.

H. Yan et al. (“Altered Effective Connectivity of Hippocampus-Dependent Episodic Memory Network in mTBI Survivors”) examined the altered effective interaction in mild traumatic brain injury (TBI) survivors' episodic memory network. Results presented that mild TBI induced increased bilateral and decreased ipsilateral effective connectivity in the episodic memory network compared with normal controls. This study provided some evidence to note the overrecruitment of the right anterior PFC caused dysfunction of the strategic component of episodic memory, which caused deteriorating episodic memory in mTBI survivors.

Z. Guo et al. (“Ipsilesional High Frequency Repetitive Transcranial Magnetic Stimulation Add-On Therapy Improved Diffusion Parameters of Stroke Patients with Motor Dysfunction: A Preliminary DTI Study”) aimed to evaluate the effects of high frequency repetitive transcranial magnetic stimulation (HF-rTMS) on stroke patients with motor dysfunction and to investigate the underlying neural mechanism. Fifteen stroke patients were assigned to the rTMS treatment (RT) group and conventional treatment (CT) group. RT group showed better improvement in motor scale and enhanced diffusion metrics in the posterior limb internal capsule, which was associated with motor functions.

S. Zhang et al. (“Alternations in Cortical Thickness and White Matter Integrity in Mild-to-Moderate Communicating Hydrocephalic School-Aged Children Measured by Whole-Brain Cortical Thickness Mapping and DTI”) explored the cortical thickness and white matter integrity following mild-to-moderate hydrocephalic in children. They found that decreased cortical thickness in the left middle temporal and left rostral middle frontal gyrus in these children compared with normal controls, and diffusion metrics were also decreased in the right body part of the corpus callosum. This study provides the evidence that structural brain changes can be used to monitor long-term outcomes and follow-up in the mild-to-moderate hydrocephalic of children.

Finally, N. Ilves et al. (“Resting-State Functional Connectivity and Cognitive Impairment in Children with Perinatal Stroke”) investigate the dysfunctions in the large-scale resting-state functional networks following perinatal stroke in children and its association with congenital hemiparesis and neurocognitive deficits. Results indicated that there were no differences in severity of hemiparesis between the periventricular venous infarction (PVI) and arterial ischemic stroke (AIS) groups. A significant increase in default mode network connectivity (FDR 0.1) and lower cognitive functions were found in children with AIS compared to the controls and the PVI group. The children with PVI had no significant differences in the resting-state networks compared to the controls and their cognitive functions were normal. They further inferred that changes in the resting-state networks found in children with AIS could possibly serve as the underlying derangements of cognitive brain functions in these children.

Great progress has been made and demonstrated the role of functional connectivity and structural abnormalities underlying brain injury (i.e., stroke, traumatic brain injury, and epilepsy), while there is also unclarity about whether these brain changes can provide a clue (as a biomarker) to establish prognostic models, develop focused treatments, and stratify patients for interventional trials. U. Horn et al. (“MRI-Biomarkers for Hand-Motor Outcome Prediction and Therapy-Monitoring following Stroke”) conducted a comprehensive review about MRI-biomarkers on the evaluation of corticospinal integrity and functional recruitment of motor resources. Compared with the functional connectivity parameters, corticospinal integrity evaluation using structural imaging showed robust and high predictive power for patients with different levels of impairment. They further suggested a combination of different measures in an algorithm to classify fine-graded subgroups of patients, because the best therapy approaches will become feasible as the subgroup become more specified.

This special issue provides promising evidence to elucidating neural plasticity associated with a wide range of brain injuries. These articles may enhance further research into examining whether the neuroprognosis of imaging biomarker is beneficial to treatment selection and effectiveness evaluation following brain injury.

*Lijun Bai* *Lin Ai* *Kevin K. W. Wang*

